# Understanding medical student evidence-based medicine information seeking in an authentic clinical simulation

**DOI:** 10.5195/jmla.2020.875

**Published:** 2020-04-01

**Authors:** Joey Nicholson, Adina Kalet, Cees van der Vleuten, Anique de Bruin

**Affiliations:** Vice Chair for Education; Head, Education and Curriculum Support; Associate Curator; and Coordinator, Systematic Review Services, NYU Health Sciences Library, New York University School of Medicine, New York, NY, Joseph.Nicholson@med.nyu.edu, http://orcid.org/0000-0001-8658-5879; Director of the Kern Institute, Medical College of Wisconsin, Milwaukee, WI, akalet@mcw.edu, https://orcid.org/0000-0003-4855-0223; Professor of Education, School of Health Professions Education, Maastricht University, Maastricht, Netherlands, c.vandervleuten@maastrichtuniversity.nl, https://orcid.org/0000-0001-6802-3119; Professor of Self-Regulation in Higher Education, School of Health Professions Education, Maastricht University, Maastricht, Netherlands, anique.debruin@maastrichtuniversity.nl, https://orcid.org/0000-0001-5178-0287

## Abstract

**Objective:**

Evidence-based medicine practices of medical students in clinical scenarios are not well understood. Optimal foraging theory (OFT) is one framework that could be useful in breaking apart information-seeking patterns to determine effectiveness and efficiency of different methods of information seeking. The aims of this study were to use OFT to determine the number and type of resources used in information seeking when medical students answer a clinical question, to describe common information-seeking patterns, and identify patterns associated with higher quality answers to a clinical question.

**Methods:**

Medical students were observed via screen recordings while they sought evidence related to a clinical question and provided a written response for what they would do for that patient based on the evidence that they found.

**Results:**

Half (51%) of study participants used only 1 source before answering the clinical question. While the participants were able to successfully and efficiently navigate point-of-care tools and search engines, searching PubMed was not favored, with only half (48%) of PubMed searches being successful. There were no associations between information-seeking patterns and the quality of answers to the clinical question.

**Conclusion:**

Clinically experienced medical students most frequently relied on point-of-care tools alone or in combination with PubMed to answer a clinical question. OFT can be used as a framework to understand the information-seeking practices of medical students in clinical scenarios. This has implications for both teaching and assessment of evidence-based medicine in medical students.

## INTRODUCTION

Evidence-based medicine (EBM) is widely accepted as the best practice in integrating research evidence into clinical decisions for the best possible patient care [1]. However, there are many barriers to physicians implementing EBM. The two most frequently reported barriers are lack of time and information overload [2–5]. For medical students to become excellent physicians, educators must ensure that the students learn how to manage and apply the voluminous information available through searching and navigating the information landscape efficiently and appropriately. Studies in this area have the potential to improve the quality of EBM education and, as a consequence, clinical care.

Recent studies of medical students’ information seeking focus on what resources they report using, what barriers to practicing EBM they perceive, and what technologies they use to perform this work [6–9]. Time management and lack of technical expertise are two of the most common challenges that medical students face when they seek research evidence to support their clinical decisions in the clinical context [6]. However, these studies do not give any insight into medical students’ abilities to successfully navigate through preferred resources in a limited time. Understanding modern information-seeking behaviors of medical students in the context of their EBM training is a first step toward addressing these significant barriers. To the authors’ knowledge, there have been no studies of how medical students spend their time on or go about navigating through multiple information resources. Understanding how our students actually approach EBM will inform and refine our EBM curriculum and enable us to audit relevant EBM behaviors and provide structured, actionable feedback in clinical settings to enhance their learning.

Two related theories inform this study: information foraging theory (IFT) and optimal foraging theory (OFT). IFT, mainly developed by Peter Pirolli, is a framework used to explain search behavior in relation to the search environment [10]. IFT uses the concepts of gain (i.e., relevant results) and cost (i.e., time or money) in a highly contextualized way specific to the cognitive task to explain and describe behavior. OFT also uses the concepts of gain and cost but specifically focuses on how to optimize the spending of time (i.e., cost) to increase the success of finding relevant information (i.e., gain). Initially developed in evolutionary biology, OFT was first applied in the modern information management environment by Sandstrom, as well as Pirolli and Card, in the 1990s to explain user search behaviors [11, 12].

In Pirolli and Card’s refinement of this theory, the patch model of foraging is analogous to information seeking [11]. In the patch model, specific resources are different patches that could be used to retrieve the needed information, and searchers must decide which patch, or resource, is likely to be most profitable at first and decide when it is time to move on to a new patch, if they have found all information they need or the patch is no longer worth their time. Using this model, Pirolli and Card examined efficiency of time spent on task and effectiveness of navigation patterns. Specifically, they described strategies that optimize information seeking through trade-offs employed between minimizing time taken and maximizing answer reliability.

While this framework has not been used to examine medical student information seeking, it has been used with clinicians in an EBM context [13]. We believe OFT is particularly well suited as a framework from which to understand and describe medical student information-seeking behaviors.

The aim of this research was to understand patterns of information seeking among senior medical students when answering a clinical question. Specifically, this study aimed to answer the following research questions:

- What sources do senior medical students use when answering a clinical question?
- What navigation patterns do medical students use to arrive at answers to clinical questions?
- Are certain navigation patterns associated with higher-quality answers?

## METHODS

To study the information-seeking behaviors of senior medical students, we used screen-captured recordings of an activity that took place as a part of “Night-onCall.” Night-onCall is a multi-station simulation experience in an Objective Structured Clinical Examination (OSCE) format. Using the thirteen “Core Entrustable Professional Activities for Readiness for Residency” framework proposed by the Association of American Medical Colleges, Night-onCall was designed to assess and address the readiness for internship of medical students who are near to graduating. All activities of Night-onCall were designed around four clinical cases [14].

### Setting

We conducted this study in 2016 using participants from the New York University (NYU) School of Medicine at the New York Simulation Center. Prior to this study, all students participating in this experience received approximately sixteen hours of instruction in EBM (Table 1) as a part of the standard medical school curriculum. The study was conducted following all participants’ EBM training at the end of their third, fourth, or fifth years.

**Table 1:** Evidence-based medicine (EBM) curriculum outline

Timing	Content	Instructional format	Outcomes assessment
Year 1: fall preclinical	“Intro to Research Questions” “Intro to PubMed and Medical Subject Headings (MeSH)”	Online module Lecture (1 hour) Hands-on workshop (1 hour)	Whole-task assignment with written formative feedback
Year 1: spring preclinical	“Intro to Evidence-Based Medicine (EBM)” “Intro to Critical Appraisal”	Lecture series (3 hours)	No assessment
Year 2: fall preclinical	“Clinical Question Formulation” “Intro to Other Databases” “Critical Appraisal”	Lectures (5 hours) Problem-based learning (PBL)–style seminars (3 hours) Hands-on workshop (1 hour)	Assessing Competency in EBM (ACE) tool; multiple-choice exam
Year 2: winter clinical orientation	“Intro to Clinical Information Sources”	Lecture (1 hour)	No assessment
Year 2–3: clinical clerkships	“EBM in Practice” (whole task)	Asynchronous video lecture (1 hour)	Whole-task assignment with written formative feedback and score

### Subjects

We recruited clinically experienced medical students for this study by email. After volunteering, students were provided a link to sign up for a time slot to participate and emailed background information on the research project.

Participation in Night-onCall was entirely voluntary, written informed consent for observation was obtained, and a financial incentive was provided. Approval for this study was obtained from the NYU School of Medicine Institutional Review Board (#i14-00867).

### Procedure and materials

Following a simulated patient case requiring students to assess an actor portraying a hospitalized patient with new-onset hypertension and headache, students were seated at a computer with Internet access with ten minutes to complete a self-paced activity. This activity provided the following clinical question: In a patient with hypertension urgency, what is the safest treatment strategy? This question arose in the interaction with the preceding simulated patient. Students were required to answer the question using evidence from the literature. They were given no further instructions as to where or how to start finding this evidence. Following their search process, they were required to explain via a free-text-entry box how they would proceed to treat the patient, based on the evidence they found. A computer program, LogMeInâ, allowed the activity on the computer screen to be viewed in real time and recorded for analysis. This performance activity was designed to reflect a “typical” clinical encounter, including time pressures and available resources.

### Analysis

The patch model of OFT was used to define the variables for extraction and analysis of the results. This model was previously used in a self-report format for clinicians [13]; here, we adapted the variables to fit direct observation of medical students. Patches are the various sources where one might forage for information, and the forager must decide when they have enough information to complete the task and move on or have exhausted one source, still need more information, and must spend time in another patch. This model allowed us to describe which resources are the most efficient (i.e., time spent finding an answer) and which navigation patterns are the most effective (i.e., selecting better sources initially).

The variables extracted from screen recordings by one coder (author Joey Nicholson) were original sources consulted, follow-up sources consulted (open text), time spent per source, total time spent on task (seconds), and success or failure in a source (dichotomous yes/no). Sources were counted only when they were being searched or their unique features were being used. For example, if a student used Google to find a citation and was taken to PubMed to read it, this was not counted as a PubMed search. Rather, PubMed was only counted when students used it to perform a search or to use its features, such as seeing related articles. Success was defined as finding any information in the source, and failure was defined as being unable to find any information in the source. Independent variables included the student’s class year and the quality of the written responses.

Quality of the written responses to the patient case were assessed on a 5-point Likert-type scale. Because there was no predefined correct answer, the quality rating scale was developed by two clinicians (Lynn Buckvar-Keltz and Ian Fagan), based on reading the student responses and categorizing the quality-defining features of the responses (Table 2).

**Table 2:** Quality rating criteria

Quality score	Criteria
5	Considered both urgent and emergent hypertension; gave both specific treatments and blood pressure targets.
4	Considered both urgent and emergent hypertension; gave either specific treatments or blood pressure targets, but not both.
3	Considered either urgent or emergent hypertension, but not both; gave both specific treatments and blood pressure targets.
2	Considered either urgent or emergent hypertension, but not both; gave either specific treatments or blood pressure targets, but not both.
1	Failed to provide a rational answer to the question or plagiarized.

To derive quality scores for each student, this rating scale was subsequently applied by 3 clinicians (author Adina Kalet, Ruth Crowe, and Verity Schaye) to all 72 cases. We used the average score of the 3 raters as the measure of quality for these written responses (intraclass correlation coefficient [ICC], 0.724; 95% confidence interval [CI], 0.59–0.81). ICC estimates and their 95% CIs were calculated using SPSS statistical package, version 24 (SPSS, Chicago, IL), based on an average-measures (*k*=3), consistency, 2-way mixed effects model.

To answer the first research question, we presented descriptive statistics that were appropriate to the data of which sources were used and in what order they were used. To answer the second question, the OFT framework was used to describe and categorize the patterns of searching in terms of success or failure in a resource, time spent, and overall navigation patterns. To answer the third question, associations between the navigation patterns and the quality of the written responses were tested using one-way analysis of variance (ANOVA). *P*-values <0.05 were considered statistically significant.

## RESULTS

### Subjects

A total of 89 students registered to participate in this study out of 327 eligible and invited students. The 89 students included 35 in the third year of the 4-year program (standard US program), 12 in the third year of the 3-year program (a highly selective, accelerated curriculum track), 36 in the fourth year of the 4-year program (standard US program), and 6 in the fifth year of the 4-year program (taking an additional year to complete a master’s degree as well as a medical degree [MD]). Of the 89 students who participated in Night-onCall, 72 were included in the final sample for this analysis. Twelve had been erroneously given an incorrect question to answer during this activity, 1 did not search for anything, 1 had a computer malfunction, and 3 had no recordings available.

### What sources do senior medical students use when answering a clinical question?

Half of students (n=37, 51%) searched only 1 source before answering their clinical question. The remainder of students mostly stopped after a second source (n=30, 42%), but a few searched a third source (n=4, 6%), and 1 searched 4 sources (1%).

Across all 72 videos, 113 searches were performed across 10 unique resources. Based on the general purpose and functionality of each resource in clinical information seeking, they were grouped into 4 main categories: point-of-care tools, PubMed, search engines, and other scholarly tools (Table 3).

**Table 3:** Source type categories and included sources

Source type category			
Point-of-care tools	PubMed	Search engines	Other scholarly tools
UpToDate	PubMed.gov	Google	Google Scholar
Epocrates	PubMed for Handhelds	Browser search bar	Web of Science
Medscape		Bing	
MedKit (a New York University–developed meta-search tool)			

**Figure 1:**
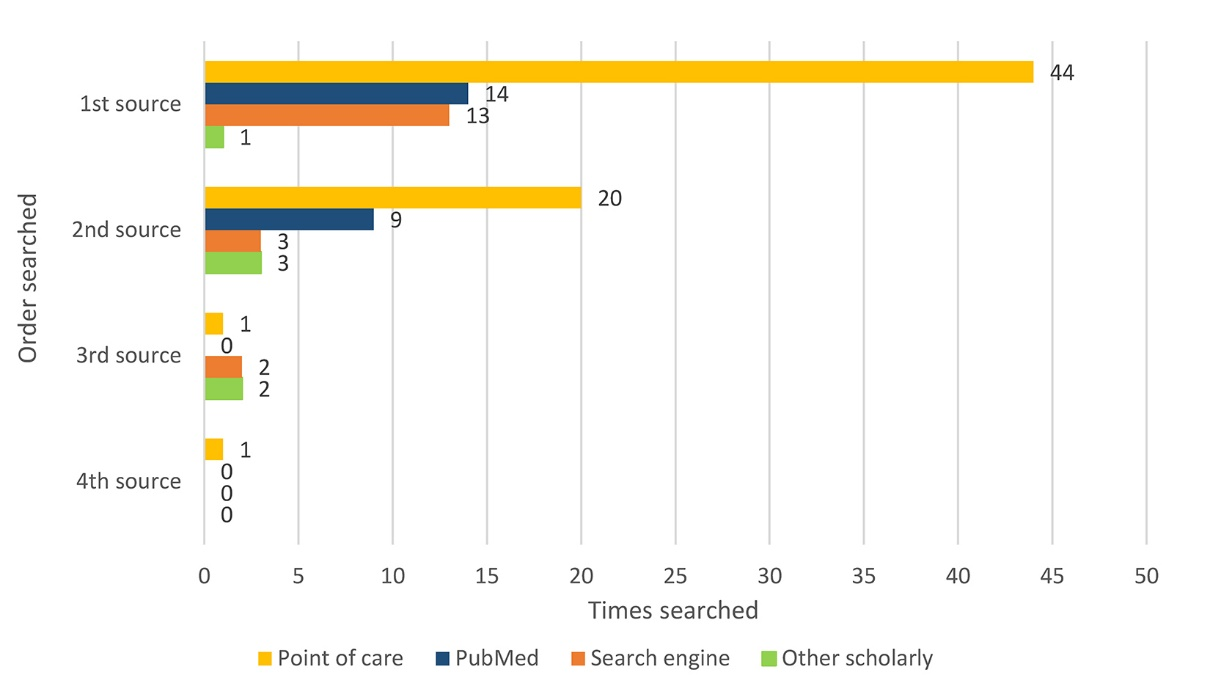
Types and order of information sources consulted

Figure 1 displays the number, type, and order of sources used. Two main source types were found to be the most used of the 4 categories. Point-of-care tools were the most frequently used sources overall (n=66/72, 58%) and the most frequently used first (n=44/72, 61%) or second (n=20/35, 57%) sources. PubMed was the second most frequently used source overall (n=23/72, 32%) and the second most frequently used first (n=14/72, 19%) or second (n=9/35, 26%) source.

### What navigation patterns do medical students use to arrive at answers to clinical questions?

Four main navigation patterns emerged from these observations. The most common pattern was the use of only point-of-care tools (n=31, 43%), followed by the use of both point-of-care tools and PubMed (n=15, 21%), the use of point-of-care tools and either search engines or other scholarly tools (n=14, 19%), and no use of any point-of-care tools (n=12, 17%).

### What time do medical students spend searching and what search success do they have?

All students were given 10 minutes to complete this activity. Time spent per search was highly variable, depending on the order of the search and the type of resources searched. Due to their skewed distribution, data for time spent searching are presented as median and inter-quartile range (IQR). Students completed their searches with a median time of 6:35 and an IQR of 5:17 to 8:11 minutes. Success in searching a source was defined as arriving at any answer.

Out of 72 first searches, 58 (81%) were successful, with a median time spent of 5:40 (IQR, 3:50–7:01). Fourteen (19%) of the first searches were unsuccessful, with a median time spent of 0:29 (IQR, 0:14–1:27). Of the 58 students who were successful, 21 (36%) chose to continue to use a second source, but the majority (n=37, 64%) chose to stop after searching 1 source. All 14 students who were unsuccessful with their first source continued to a second source, leaving 35 (49%) students who continued to a second source.

Out of 35 second searches, 29 (83%) were successful, with a median time spent of 5:35 (IQR, 2:57–7:18). Six (17%) of the second searches were unsuccessful, with a median time spent of 0:40 (IQR, 0:26–0:44). Of the 29 students who were successful, 4 (14%) chose to continue to use a third source, but only 1 (17%) of the 6 who were unsuccessful chose to continue to a third source.

Out of the 5 searches using a third source, 4 (80%) were successful, with a median time spent of 3:05 (IQR, 1:35–4:14). One (20%) of the searches was unsuccessful and lasted 0:27. This unsuccessful searcher was the only searcher to continue to a fourth source. The single fourth source search was successful and lasted 2:55.

Median time spent on unsuccessful searches was consistently lower than median time spent on successful searches. Rates of conducting a subsequent search dropped after a searcher had conducted at least one successful search. Table 4 summarizes the median search times and percent choosing to pursue a subsequent search for both successful and unsuccessful searches.

**Table 4:** Information searching time and decision to use a subsequent source (median and inter-quartile range [IQR])

Source	Successful searches					Unsuccessful searches				

	Search time	min:sec	n	Subsequent source used	n	Search time	min:sec	n	Subsequent source used	n
First	5:40	3:50–7:01	58	36%	21	0:29	0:14–1:27	14	100%	14
Second	5:35	2:57–7:18	29	14%	4	0:40	0:26–0:44	6	17%	1
Third	3:05	1:35–4:14	4	—	0	0:27		1	100%	1
Fourth	2:55		1							

### What are the search outcomes and how efficient are the searches by resource type

Search time and success both varied widely by resource type. Students had the highest success rates when they used other scholarly tools (100%) or point-of-care tools (96%). Conversely, they had lower success rates when they used either search engines (68%) or PubMed (48%). Success rates for a resource type were not related to the efficiency of a resource (defined as average time spent on successful searches divided by the number of successful searches). The most efficient resource type was search engines, averaging 3:28 minutes to find an answer. The least efficient resource type was PubMed, averaging 6:53 minutes to find an answer. Table 5 summarizes search time, success rates, and search efficiency.

**Table 5:** Search time, success, and efficiency by type of information source (all searches combined)

Source type	Search time		Search success		Average search efficiency
	min:sec	n	%	n	min:sec
Point-of-care tools	5:58±3:52	66	96%	63	5:45
PubMed	4:20±6:54	23	48%	11	6:53
Search engines	1:47±2:13	18	68%	13	3:28
Other scholarly tools	4.91±7.83	6	100%	6	5:56

### Are certain navigation patterns associated with higher-quality answers?

**Figure 2:**
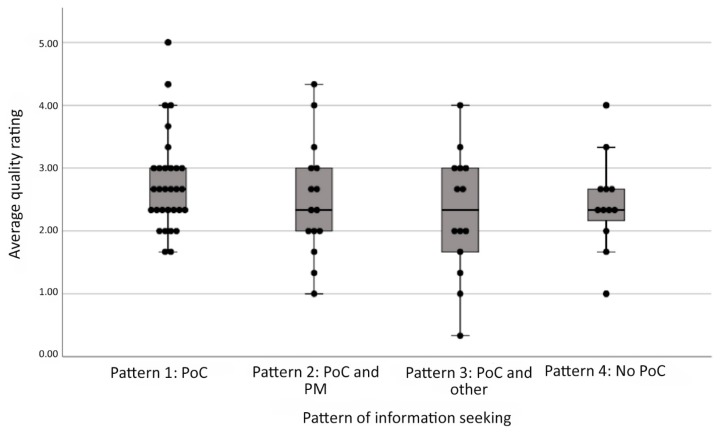
Average quality ratings per information seeking pattern PoC: point-of-care tools; PM: PubMed; Other: search engines or other scholarly sources; No PoC: no inclusion of point-of-care tools.

The quality ratings of students’ answers to the clinical question, which could range from 0 to 5, were approximately normally distributed, with an overall average quality rating of 2.57 (standard deviation, 0.855). There was no statistically significant difference among the 4 navigation patterns in the average quality ratings of written responses to the clinical question (*F*(3,68)=1.228, *p*=0.306). The highest average quality ratings were for students who exhibited pattern 1, which was use of point-of-care tools alone (2.77±0.78). The lowest average quality ratings were for students who exhibited pattern 3, which was use of point-of-care tools and either search engines or other scholarly tools (2.29±0.99). Pattern 1 students tended to have higher-quality answers than pattern 3 students, although this difference was not statistically significant (*p*=0.07). Figure 2 summarizes the average quality ratings and ranges for each navigation pattern.

## DISCUSSION

In this study, we operationalized the patch model of OFT by documenting the effectiveness of first and subsequent patch selection and efficiency in terms of time spent finding an answer. We found that when seeking answers to a particular clinical question in a simulated environment, most medical students who were near to graduating showed relatively effective navigation patterns toward trustworthy information sources, and many were able to extract from those sources a high-quality written answer to the clinical question. All students used at least one reliable source of medical information (i.e., point-of-care tool, PubMed, or other scholarly tool) to find a defensible answer to the clinical question. While students conducted 16% of all searches initially in a search engine, which is not considered a trusted source, they consulted reliable sources in the full search process. This indicated that students were able to effectively identify the most reliable sources, possibly through a combination of their explicit curriculum and their clinical experience of what worked best.

Most students minimized the time they spent on unhelpful resources and quickly moved to the next more fruitful source in their quest for an answer. This is another sign of an effective information seeker. However, the high average search time for the three reliable sources suggested that there was room for improvement in search efficiency in each of these sources. The process in OFT through which better efficiency can be achieved is called enrichment, which is when an information seeker utilizes the tools built into each database to navigate through that source to more quickly obtain an answer. For PubMed, this could mean using the Clinical Questions filters to limit to relevant study designs. In UpToDate, this could mean using the side navigation links to go to relevant sections of the article without reading or scrolling through the entire article.

The quality of answers to the clinical question varied widely, reflecting a range in the students’ ability to rapidly interpret and apply the evidence to a patient. We had an a priori expectation that more effective and efficient navigation patterns might be directly related to the quality of the answers for the clinical question. Instead, we found no statistically significant difference among the four different patterns, although those who used point-of-care tools alone had slightly higher-quality written responses than those who started with point-of-care tools and then switched to multiple other sources. Although it was possible that concentrating on a highly curated and easily applied source led to a better answer, it was also possible that students with higher-quality answers might have had experience with the clinical question and were merely confirming what they already knew from experience.

Future studies should investigate the associations and pathways between being able to locate and navigate through a reliable source of evidence and the application of that evidence to patient care. This could be researched by having participants talk through their decision-making processes as they navigate through resources or explain their answers and decision-making process after the search. Also, it would be important to control for prior experience with the particular clinical question and explore EBM practices across clinical content.

### Optimization

This is the first study to use OFT to describe and understand the information-seeking behaviors of medical students. With a focus on effectiveness and efficiency as collected through source type selection and time to arrive at an answer using those sources, OFT is well suited to analyze and interpret medical students’ information seeking. These same data elements were used in Slawson, Shaughnessy, and Bennett’s work on patient-oriented evidence, which used the equation “Usefulness of Medical Information=(relevance×validity)/work” [15]. Their work posited that to be most effective in managing information in a clinical context, one must start with the most relevant sources that take the least time. The data elements of source type selection and timing could be used in the future to help frame instruction and design assessment instruments around real-world behaviors instead of idealized information-seeking behaviors.

While, in general, the students appeared to be good at identifying reliable sources and then moving on when they were not successful, there was still opportunity for optimization of their information seeking. The success rate for point-of-care tools was nearly perfect, but this was not the case for PubMed, which is much more likely to be influenced by an individual’s searching skill level. PubMed searching is consistently taught throughout the curriculum, and despite the fact that students remain aware of the high reliability of this resource, clearly there is room for improvement. Current teaching strategies for PubMed in medical school curricula focus mostly on more traditional lecture hall or computer lab classes [16, 17], as opposed to integration into the clinical context where students will be expected to perform these behaviors. Just-in-time teaching strategies that incorporate formative feedback need to be designed and tested.

One possibility for improving PubMed skills in a time-limited context is to provide more opportunity for embedded librarians to give feedback to students on their own searches, and teach, demonstrate, and model efficient use of this resource at the point of care. While we have used the patch model of OFT to describe information-seeking patterns, the concept of enrichment could be used so that searching in a source is optimized [11, 18]. Using concepts of enrichment in this context would entail teaching and assessing behaviors like identifying specific study designs that would best answer a clinical question or using the database’s built-in filters as a shortcut.

OFT is ideally suited to help understand and describe behaviors in EBM, where limited time and information overload are key barriers to effective practice. It also has implications for informing assessment of EBM behaviors. The current range of assessment tools for these expected behaviors are limited to knowledge and skill, as direct observation of performance has not yet been achieved [19]. Since there currently are no tools for direct observation of these behaviors, effectiveness and efficiency as operationalized in this study from OFT can be used to help frame assessments and feedback to learners in their clinical and near-clinical environments. For example, an observer might answer the questions: did they use a known reliable source?, and if so, did they use that source’s built-in filters? Answering these two questions will give an immediate sense of whether they are effective at source selection and whether they are efficient at using a source.

### Limitations

This observational study was conducted with volunteer participants from one medical school in the United States. These volunteers might have been more predisposed to practicing clinically relevant skills for their upcoming residencies and potentially performed at a different level than the general population of medical students. This was also a single clinical case, and students were provided with a clinical question to answer. Generalizability across clinical content was limited. Clinical information seeking changes rapidly as tools change, evolve, and improve. While efforts were taken to be as similar to real-life activities as possible, the current study was done in a time-limited simulated exam setting and should be interpreted with that context in mind.

As we used a common and well-known clinical scenario among hospitalized adults—new-onset hypertension with headache—there was rich and detailed information available in the point-of-care tool that applied directly to the case at hand. It is likely that novice clinicians would have performed very differently in seeking information to make a clinical decision in a less common scenario, where they would have needed to rely more on a resource such as PubMed. This needs to be confirmed because it potentially has significant impact on how to tailor curriculum and clinical decision-making support for novice clinicians. Future studies could include more complicated scenarios, talk-aloud protocols, and follow-up interviews to better understand the reasoning behind the information-seeking choices that students make and how their navigation patterns relate to their existing knowledge of a given topic and synthesis of their answer to the clinical question.

## CONCLUSION

We found that clinically experienced medical students began information seeking in a variety of resources; however, most began in either a point-of-care tool or in PubMed. While students might spend more time in one resource once they have found an article with an answer, they very quickly moved to another resource when their initial search efforts did not offer an immediate and clear answer. Students with the highest-quality responses answered the clinical question using point-of-care tools alone, but there were no statistically significant differences among navigation patterns. Using OFT to analyze and understand these patterns can help inform more effective embedded models of both instruction and assessment.

## 

**Joey Nicholson**, Joseph.Nicholson@med.nyu.edu, http://orcid.org/0000-0001-8658-5879, Vice Chair for Education; Head, Education and Curriculum Support; Associate Curator; and Coordinator, Systematic Review Services, NYU Health Sciences Library, New York University School of Medicine, New York, NY

**Adina Kalet**, akalet@mcw.edu, https://orcid.org/0000-0003-4855-0223, Director of the Kern Institute, Medical College of Wisconsin, Milwaukee, WI

**Cees van der Vleuten**, c.vandervleuten@maastrichtuniversity.nl, https://orcid.org/0000-0001-6802-3119, Professor of Education, School of Health Professions Education, Maastricht University, Maastricht, Netherlands

**Anique de Bruin**, anique.debruin@maastrichtuniversity.nl, https://orcid.org/0000-0001-5178-0287, Professor of Self-Regulation in Higher Education, School of Health Professions Education, Maastricht University, Maastricht, Netherlands
